# Changes in Sport Nutrition Knowledge, Attitudes/Beliefs and Behaviors Following a Two-Year Sport Nutrition Education and Life-Skills Intervention among High School Soccer Players

**DOI:** 10.3390/nu10111636

**Published:** 2018-11-02

**Authors:** Megan M. Patton-Lopez, Melinda M. Manore, Adam Branscum, Yu Meng, Siew Sun Wong

**Affiliations:** 1 Division of Health & Exercise Science, Western Oregon University, Monmouth, OR 97361, USA; 2 Nutrition, School of Biological and Population Health Sciences, College of Public Health and Human Sciences, Oregon State University, Corvallis, OR 97331, USA; melinda.manore@oregonstate.edu (M.M.M.); mengy@oregonstate.edu (Y.M.); SiewSun.Wong@oregonstate.edu (S.S.W.); 3 Biostatistics, School of Biological and Population Health Science, College of Public Health and Human Sciences, Oregon State University, Corvallis, OR 97331, USA; adam.branscum@oregonstate.edu; 4 Family and Community Health, School of Biological and Population Health Science, College of Public Health and Human Sciences Oregon State University, Corvallis, OR 97331, USA

**Keywords:** sport nutrition, diet behaviors, adolescent, low-income, Latino youth, soccer, sport, obesity prevention

## Abstract

The purpose of this study was to examine the impact of a sport nutrition education and life-skills intervention on sport nutrition knowledge (SNK), attitudes/beliefs and dietary behaviors relevant to sport nutrition among high school (HS) soccer players. Three assessments were done over the 2-year intervention (baseline = time 1, end year 1 = time 2, end year 2 = time 3). Participants (*n* = 217; females = 64%; Latino = 47.5%; 14.9 ± 0.9-year; 46.5% National School Breakfast/Lunch Program) were assigned to an intervention group (IG, *n* = 153; 9 schools) or comparison group (CG, *n* = 64; 4 schools) based on geographical location. Differences over time were examined based on group, sex, socioeconomic status (SES) and race/ethnicity. The IG increased SNK scores by ~10% (time 1 = 51.6%; time 3 = 60.9%; *p* ≤ 0.001), with the greatest change in the female IG vs. CG and no differences in male IG vs. CG. Daily breakfast consumption was 53.7% in both groups. IG players were 3 times more likely (95%CI = 2.59, 7.77) to report trying to eat for performance (IG = 48.7% vs. CG = 30.2%). By time 3, IG players were less likely to report that ‘diet met nutritional requirements’ (31.6%) compared to CG (47.6%). For IG, the consumption of lunch (≥5-days/week) did not change (92.2–93.4%), but declined in the CG (90.6%) (*p* = 0.04). No other differences by sub-population (race/ethnicity, SES) were observed. Our findings indicate that HS athletes are motivated to learn and improve diet behaviors, and benefit from team-based nutrition interventions. Future interventions should consider delivery of curriculum/experiential learning during a defined training period, with messages reinforced with supports at home, school and athletic settings.

## 1. Introduction

Adolescence is a time of transition [1] in which youth begin to establish life-long diet and physical activity habits. During this time, physical activity typically decreases [2,3,4], while independence in making food choices increases [5]. In addition, adolescence is typically a time of rapid growth, requiring adequate nutrition to achieve full physiological potential [6]. Thus, the diet and physical activity patterns established during these years not only impacts youth’s current health and body size, but can continue into adulthood and impact over health, weight and risk for chronic disease [7]. Research shows that adolescents have poor dietary habits and eating patterns [8] and do not meet the current dietary recommendations [9]. Thus, understanding how to design and implement age-appropriate and accessible health promotion programs to address these issues is needed [7]. To be successful, these programs must engage youth, while supporting healthy eating behaviors and a physically active lifestyle [9]. Adolescent athletes participating in high school (HS) sports represent a potential target group for interventions to promote and build these healthy life-skills and behaviors [10]. These active adolescents already know about physical activity, training hard, and working toward a goal, and are open to implementing diet behaviors that can help them improve sport performance. Why not capitalize on this interest by teaching sport nutrition education to improve eating behaviors for sport performance, health, and chronic disease prevention?

Based on 2017 data, 54.3% of United States (US) HS students participated in sports [11]. One of the most popular youth team sports is soccer, especially in Latino youth [12]. In 2016–2017, over 24,000 HS students played soccer [13]. Unfortunately, adolescent athletes do not always make healthful food choices [14,15] or have the best food options available to them at sporting events [16]. Active youth, especially those engaged in intense sport participation, have unique energy and nutritional needs [17]. Thus, the eating patterns of adolescent athletes need to meet their growth and developmental needs, and the energy and nutritional demands associated with general physical activity and sport participation [18,19,20]. Adolescent female athletes also need to meet the energy demands of menses and reproductive health [21]. Based on the 2014 Sports Dietitians Australia Position Statement on Adolescent Athletes [17], adolescents involved in sport need diets that provide adequate energy (i.e., calories), protein, carbohydrate, unsaturated fat, iron, calcium, vitamin D and fluids. Athletes who restrict energy intake to maintain a low body weight [22,23] or have diets high in processed foods [24,25] are at the greatest risk for poor energy and nutrient intakes. Female athletes are especially at risk for poor energy [22,26,27] and micronutrient intakes, including calcium [28,29,30], B-vitamins [31], zinc and iron [32]. The International Olympic Committee (IOC) consensus statement on relative energy deficiency in sport (RED-S), highlights the numerous health and performance consequences of inadequate energy intake in athletes [26]. The negative impact of restricted energy intake on bone health in female athletes, including adolescent female athletes, is well documented in the Female Athlete Triad [21,26]. One potential factor contributing to poor energy and nutrient intakes in adolescent athletes is the timing of meal consumption, both throughout the day and around sport practice or games. Consistent meal patterns may preserve lean body mass [17,33] and replenish stored carbohydrate (glycogen), which is an important fuel for exercise and brain function [17]. Skipping meals, particularly breakfast, has been identified as a concern among adolescents in general [34,35,36], but may be especially problematic for adolescent athletes. If breakfast is skipped, then only one meal opportunity will occur prior their typical early afternoon sport practice or game. This means athletes start their sport practice being inadequately fueled for intense physical activity. If adolescent athletes can learn how to fuel their body for sport and health by selecting healthy foods and appropriate beverages, they may establish and carry these diet behaviors into adulthood [37].

Research examining sport nutrition knowledge (SNK) and behaviors in adolescent athletes is primarily cross-sectional and uses club- or elite-level athletes. Generally, this research shows that adolescent athletes have low SNK (ranging from 55–74%) [38,39,40,41], and the SNK they do have does not consistently predict food choices [38]. Recently, Manore et al. [42] examined SNK, attitudes and beliefs in 535 HS soccer players. They found that SNK was low (46%), especially in Latino youth (39%) and those participating in National School Breakfast/Lunch Program (NSLP) (41%). Little research has explored SNK, attitudes or belief differences in active adolescents based on sex, race/ethnicity, or socioeconomic status (SES). Yet researchers repeatedly highlight the diet-related disparities that exist in adolescent populations based on sex [17,21,26,43,44], race/ethnicity [45], and SES [46]. Finally, based on population census data, it is projected that youth of color will represent more than 50% of the US adolescent population by 2060 [47]. Thus, there is a need for researchers to include more diverse youth when examining sport nutrition issues in adolescent athletes, especially adequate energy and intake of nutrient dense foods [27,48].

Intervention programs focused on nutrition or behavior change in the adolescent athlete population are also limited. Available interventions have focused primarily on improving disordered eating, body image [10] and restrictive dietary behaviors [49] among female athletes, reducing alcohol and sport supplement use among male athletes [50], and improving hydration in active youth [51,52]. These interventions ranged from 1 day to 8 weeks. Only three interventions have targeted changing SNK and dietary practices in adolescent athletes [53,54,55]. However, these studies all lacked a control group, had small sample sizes (*n* = 11–49), focused on individual sports (swimming, cycling, sailing, etc.), and varied in duration and how SNK scores were reported and measured. Walter et al. [55] focused on training undergraduate nutrition student to work with HS athletes and reported no changes in SNK and behaviors. To date, no team-based interventions have focused on changing SNK and behaviors in HS athletes, beyond changing hydration practices. Capturing adolescents while they are still active and engaged in youth sports provides a ‘window of opportunity’ to cultivate life-skills that support life-long health and obesity prevention, such as healthy eating behaviors, meal planning, grocery shopping and cooking skills. This is also an opportunity to teach youth athletes how to fuel and hydrate their body for sport, physical activity and health, and to discern if sport foods and supplements are needed.

The WAVE~Ripples for Change: Obesity Prevention in Active Youth (WAVE) intervention program was developed for HS soccer players to teach sport nutrition and life-skills (e.g., meal planning, shopping on a budget, food preparation/cooking skills, and gardening) to support sustainable healthy eating and to encourage physical activity outside of sport. Thus, the purpose of this 2-year study was to examine the impact of a sport nutrition education and life-skills intervention on SNK, attitudes/beliefs and behaviors among HS soccer players and to determine differences in outcomes based on sex, SES and race/ethnicity.

## 2. Materials and Methods

### 2.1. WAVE Program Overview

**Figure 1:**
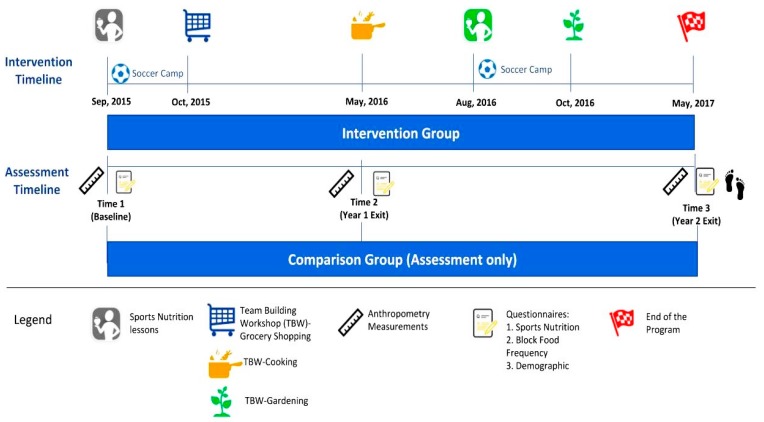
The WAVE program intervention experimental design (2015–2017), specific for sport nutrition knowledge, attitudes/beliefs and behaviors and life-skills data. TBW, team-building workshop.

The WAVE program was a 2-year integrated (research, education, and extension) obesity prevention intervention targeting HS soccer players (aged 14–19 years). The intervention was age-specific and included health assessments, face-to-face sports nutrition lessons, experiential learning and team-building workshops (TBWs). The WAVE educational objectives were to teach life-skills (e.g., meal planning, shopping on a budget, food preparation/cooking skills, and gardening) and sports nutrition education to support sustainable healthy eating among HS soccer players. Further details of the larger study can be found elsewhere [42,56,57,58]. Eligibility criteria included: (1) age 14–19 years; (2) enrolled in HS soccer; (3) living with a parent/caregiver; (4) no medical conditions preventing a normal diet; (5) internet access during the 2-year study; and (6) proficiency in English. Figure 1 shows the experimental design of WAVE program for this manuscript only, see the full WAVE experimental design in Supplement Figure S1. Here we present the 2-year intervention data for changes in SNK, attitudes/beliefs and behaviors for participants who completed all questionnaires at each of three time points. The WAVE program and follow-up evaluations were approved by the Oregon State University (OSU) Institutional Review Board (#6317).

### 2.2. Recruitment and Participants

We used a multi-step recruitment process for this study. First, soccer coaches and their schools were recruited through OSU 4-H Soccer Program. Second, soccer players were recruited through their coaches. Third, parents were recruited at soccer parent meetings. Soccer teams were then assigned (non-randomized) to either intervention (*n* = 278; 9 schools) or comparison (*n* = 110; 4 schools) group based on geographical location within the Willamette Valley, Oregon.

**Figure 2:**
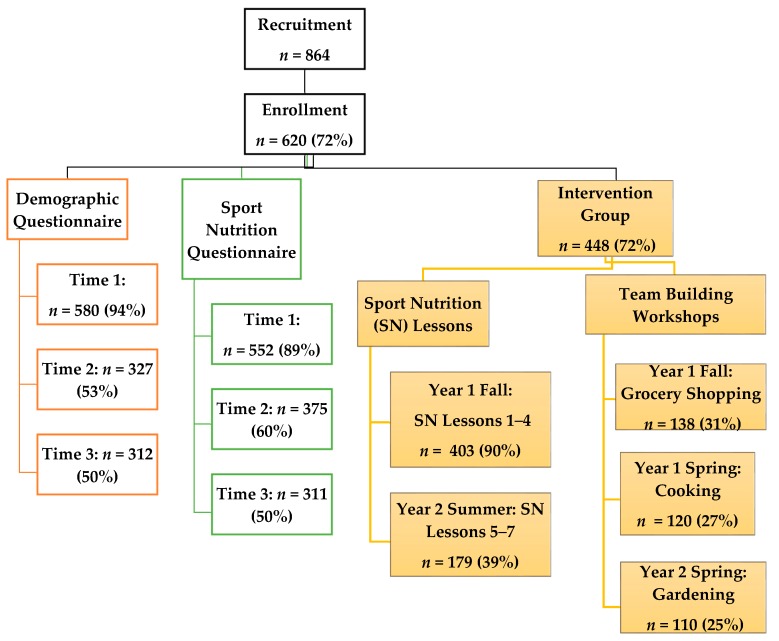
WAVE program youth participant attendance and retention for sport nutrition (SN) related questionnaires and activities.

The WAVE project recruited 864 HS soccer players with 72% (*n* = 620) enrolled and submitting youth assent and parent consent forms [59]. Figure 2 describes WAVE program youth participant attendance for each sport nutrition related activity for the 2-year period. For this manuscript, participants were included if they completed two questionnaires (described below) at baseline (time 1), end of year 1 (time 2) and end of year 2 (time 3) (*n* = 217; 14–18 years; 13 schools, 24 soccer teams). Only the intervention group (*n* = 153 (IG)) received all aspects of the intervention, which is described in Figure 1. The comparison group (*n* = 64 (CG)) also completed all assessment activities, but did not receive the intervention. Overall, the participants (*n* = 217) for this study were 64.0% female, 71.4% in grades 9 and 10, predominately Latino (47.5%) and White (44.2%), and reported playing soccer an average of 6.9 years. As a group, 46.5% participated in NSLP and 65.4% reported no sport injuries during the last 12-months.

### 2.3. Assessments and Questionnaires

All participants completed the questionnaires either at school or while attending soccer camp. WAVE participant’s data were collected using two questionnaires: (1) a demographic, health history and sport experience questionnaire designed for the study, including questions regarding number of years playing soccer, sport related injuries, and general diet behaviors; and (2) a validated SNK questionnaire [39]. Participants also indicated their participation in the NSLP, which was used as a proxy for low household income [23,24]. The SNK questionnaire had been previously validated in HS rugby players in Ireland [39]. The questionnaire consisted of 40 questions, including questions regarding training schedule. Table 1 outlines the key themes and topic areas addressed in the sport nutrition questionnaire. A SNK score was calculated for each athlete by adding the total number of correct questions (*n* = 10) covering four domains (hydration, protein/carbohydrate, supplements and pre/post exercise food selection). The SNK questionnaire was administered in the presence of the researchers to minimize discussion of responses between participants, and no members of the coaching staff were present. Cronbach’s alpha was used to estimate internal reliability for the nutrition knowledge subsection, which was determined to be 0.51. Baseline results for the SNK, and attitudes and behaviors related to sport nutrition for WAVE participants can be found at Manore et al. [42].

### 2.4. Intervention

The 2-year intervention included sport nutrition lessons, experiential learning in the classroom, and TBWs, which were delivered to IG soccer teams during fall soccer season and summer camps. The WAVE HS sports nutrition curriculum [60] was delivered to IG teams and coaches by a registered dietitian nutritionist (RDN), trained in sports nutrition and who had played collegiate/professional soccer prior to the intervention, all lessons were pilot-tested and revised based on input from athletes and sports nutrition experts (RDNs, Certified Specialist in Sport Nutrition (CSSD)). Topics covered were as follows: hydration; pre/during/post-exercise fueling; body composition/image; maintaining muscle and staying healthy; and eating well while dining out (see Table 2). Lessons involved experiential learning such as role playing, cooking demonstrations and food tastings, meal and snack planning around training and games. Newsletters reinforced lessons and provided recipes/tips to meet sports fuel and nutrition needs. Three life-skill training sessions were delivered to teams via the TBWs (~1–1.5 h each) and focused on meal planning, shopping on a budget, food preparation/cooking skills, and gardening [58]. See Figure 1 for WAVE Program timeline.

### 2.5. Statistical Analysis

The statistical analysis was completed in two phases. Phase one investigated the differences between those who completed all aspects of the intervention (*n* = 217; *completers*), and those lost to follow up (*n* = 340; *incompleters*). Phase two examined the difference between the groups (IG, *n* = 153; CG, *n* = 64) over time (3 time points) on SNK, attitudes and beliefs, and behaviors.

The outcomes (sociodemographic, psychosocial and behavioral variables) for participants who completed the 2-year intervention (*n* = 217; *completers*) were compared to those lost to follow-up (*n* = 340; *non-completers*). We used multivariate analysis to examine retention probability. Results showed that participants assigned to the IG were 2 times more likely to complete the 2-year intervention compared to CG peers (Odds Ratio (OR) 2.02, 95% Confidence Interval (CI) 1.29, 3.18, *p* = 0.002). Furthermore, females were 1.7 times more likely to participate throughout the intervention compared to males (OR 1.67, 95% CI 1.14, 2.46, *p* = 0.009). Participants in the 12th grade at baseline were less likely to complete the intervention compared to 9th-grade participants at baseline (OR 0.036, 95% CI 0.01, 0.10, *p* = 0.001), since 12th graders graduated before the intervention was over. No other differences based on socio- demographic factors was observed between those who completed the full protocol and those who did not. Daily breakfast was the only outcome variable (e.g., SNK, attitudes/beliefs, behaviors) that differed between completers and non-completers. At baseline, completers were 1.5 times more likely to report eating breakfast daily compared to non-completers (OR 1.56, 95% CI 1.05, 2.33, *p* = 0.000). Successful completion of the program did not depend on geographical location. All participants were recruited from two counties. Retention among the counties was similar (39.2% vs. 39.0%; X^2^ = 0.0046, *p* = 0.95).

For all participants (*n* = 217), descriptive statistics (mean, standard deviation (SD)) were calculated for baseline demographic characteristics. Independent *t*-tests were used to examine differences between IG and CG on age, number of years playing soccer, age started preparing meals and SNK scores. Chi-squared tests were used to examine differences between groups (IG, CG) for discrete variables (sex, race/ethnicity, year in school, no injuries in the past 12 months, participation in the NSLP, prepares meals for self). Chi-squared tests were also used to compare outcome variables related to attitudes, beliefs and behaviors between the groups over time.

To determine the impact of the intervention, outcome variables were compared between groups (IG, CG) over the three time periods. Mean differences between groups for variables related to SNK, attitudes/beliefs and behaviors were determined through longitudinal data analysis using generalized estimating equations. Each model contained the independent variables of time (time 1 = baseline, time 2 = end of year 1, time 3 = end of intervention; treated as a categorical variable) and group (IG or CG), and the interaction of group and time (time, group and group × time). Each model used age, race/ethnicity, sex, and SES as adjustment variables. During the 2-year study, the IG spent 4 h in sport nutrition lessons and 3 h in TBW. The impact of frequency of participation (e.g., hours) on SNK score and change score was examined with the addition of a dose variable (total hours of participation) in the model. We assumed that missing data were missing at random. All analyses were conducted using Stata SE 14.2 (College Station, TX, USA).

## 3. Results

Participant demographic characteristics are presented in Table 3. Overall, 60% of participants were female (*p* = 0.005), and 46.5% participated in the NSLP (*p* = 0.057), with more males (46.2%) than females (36%) (*p* = 0.020), and more Latinos (82%) than White (14%) (*p* < 0.0001). At baseline, the IG had played soccer longer (1.5-year longer, *p* = 0.008) than CG. Results comparing the main outcome variables at baseline between groups showed that the IG reported that athletes had different nutritional requirements (IG = 60.6% vs. CG = 41.3%; X^2^ = 6.55, *p* = 0.010), and that muscle mass is important to performance (IG = 68.3% vs.CG = 52.4%; X^2^ = 4.77, *p* = 0.029). In addition, a greater number of IG reported consuming sugar sweetened beverages prior to physical activity (IG = 68.6% vs. CG = 51.6%; X^2^ = 5.68; *p* = 0.017). No other differences between IG and CG were observed at baseline.

The WAVE program provided seven hours of nutrition education (sport nutrition lessons (4 h), TBW (3 h)) to the IG, with a mean participation of 4.1 h (SD = 1.6 h). Overall, there were no differences in participation hours based on sex, but the NSLP participants had a lower rate of participation than non-NSLP participants (3.6 h vs. 4.4 h; *p* = 0.001), and participants identified as White had higher participation (4.4 h) than Latino (3.5 h) (*p* = 0.0068).

### 3.1. Sport Nutrition Knowledge

The SNK results for each time period are presented in Table 4, with a maximum possible score of 10 at each time point. At baseline (time 1) there was no statistical difference in the sex-race-income adjusted mean SNK score between groups. However, the IG significantly increased their SNK scores by ~10% from time 1 to time 3 (*p* ≤ 0.001), while there were no changes in the CG (see Table 4). Within the IG at time 3, the final SNK scores were as follows: non-NSLP participants (64.3% vs. 54.5% NSLP), White (63.2% vs. 55.1% Latino) and males (61.1% vs. 59.1% females). The significant change over time in total SNK scores in the IG vs. CG is attributed to the significant improvement in the scores of the IG (time 1 = 51.6%; time 3 = 59.1%) vs. GC (time 1 = 44.8%; time 3 = 50.2%) female athletes (e.g., significant group × sex interaction). Among males, there was no difference in the sex-race-income-adjusted mean SNK score between the groups at any time period. For females, there was no statistical difference between IG and CG for the sex-race-income-adjusted mean SNK score at time 1. No other differences by sub-population (NSLP participation nor race/ethnicity) were observed in SNK score changes over time.

### 3.2. Attitudes and Beliefs Relevant to Sport Nutrition

Table 5 shows the attitudes and beliefs relevant to sport performance between groups. At time 1, most participants (IG = 84.5%; CG = 88.9%) reported that diet was important to performance. These responses increased to 92.1% and 93.6% by time 3 in the IG and CG, respectively. Throughout the study, a greater percentage of IG (time 1 = 60.6%; time 3 = 73.0%) players reported that athletes have different nutritional requirements than their peers compared to the CG (OR 2.00 (CI = 1.28, 3.16; *p* = 0.003). In addition, soccer players in the IG (time 1 = 68.3%; time 2 = 70.7%, time 3 = 62.2%) were twice as likely as the CG players to agree with the statement that muscle mass is important for performance (*p =* 0.002). No differences based on sex, race/ethnicity or NSLP participation status were observed.

Examination of changes in beliefs over time showed that at time 2, soccer players in the IG were twice as likely as CG players (CI = 1.20, 5.70; *p* = 0.015) to report that they try to eat for performance (IG = 46.6% vs.CG = 33.3% CG). At time 3, IG players were three times likely as CG players (CI = 2.59, 7.77; *p* = 0.002) to report that they try to eat for performance (48.7% IG vs. 30.2% IG). By time 3, soccer players within the IG (31.6%) were less likely to report that their diet met nutritional requirements than the CG (47.6%) (OR = 0.43; CI = 0.18, 0.99; *p* = 0.48). Differences over time by sex, race/ethnicity or NSLP participation status were not observed. No other statistically significant difference in changes over time were observed in attitudes and beliefs relevant to sport nutrition (see Table 5).

### 3.3. Dietary Behaviors Related to Sport Performance

Baseline values and changes over time among the five dietary behaviors are provided in Table 6. At baseline, self-report of dietary behaviors related to sport performance were similar between the groups, except for the consumption of sugar sweetened beverages (SSB) 1–4 h prior to physical activity (*p* = 0.017) (Table 6). Among the IG, 68.6% reported consuming SSB, compared to 51.6% of the CG participants, with no differences based on NSLP or sex. For the IG, the consumption of SSB 1–4 h before physical activity decreased by 17% over the intervention, but there was no time × group interaction. Over half of the participants (53.7%) reported consuming breakfast daily at time 3, regardless of group assignment (X^2^ = 0.0123, *p* = 0.912). There were no differences between groups for consumption of breakfast over time. There was a significant group × time interaction for the change in ‘eat lunch 5 or more days a week’ at time 2 *(p* = 0.027) and time 3 (*p* = 0.040). Throughout the intervention, the proportion of athletes within the IG reporting eating lunch at least 5 days per week remained at 92.2–93.4%, while the percentage of CG participants reporting this behavior decreased from 98.4% (time 1) to 90.6% (time 3). There were no group or time differences for fueling behavior before/after physical activity. For the IG group, eating within 1 h of physical activity increased by 10% from time 1 (33.1%) to time 3 (43.7%), while there was no change in the CG (time 1 = 38.7%; time 3 = 33.3%); however, the differences in change over time between groups were not statistically significant when controlled for sex, race/ethnicity and NSLP participation status. For both groups, the proportion of participants reporting ‘eat within 1 h after physical activity’ did not significantly change across the intervention (71–79%) (see Table 6).

## 4. Discussion

This is the first study to assess the impact of a sports nutrition and life-skills intervention on SNK, dietary attitudes/beliefs and dietary behaviors among a diverse group of HS team-sport athletes, stratified by sex, participation in NSLP, and race/ethnicity. High school soccer players participated in a 2-year integrated (research, education, and extension) physical activity, sport nutrition, and family consumer science life-skills intervention and completed data collection activities at three time points. Overall, the intervention significantly improved total SNK scores, especially among female athletes, increased the athlete’s awareness of their nutritional needs and the motivation to eat for performance, and supported eating behaviors related to daily lunch consumption and less SSB used. Changes over time among the IG were similar across the sub-population groups (sex, race/ethnicity, SES), with the exception of SNK. We found no changes in self-reported consumption of daily breakfast, pre/post-exercise fueling behaviors, or attitudes/beliefs regarding sport supplement use.

### 4.1. Changes in Sport Nutrition Knowledge

Mean SNK scores among the IG significantly increased by 10% and were statistically higher than that of the CG at time 2 and 3. Examination of the outcomes by sex revealed that the IG females had a significant change over time in SNK (~10% higher) (time 1 = 52%, time 2 = 60%) compared to females in the CG (time 1 = 45%; time 3 = 50%). At time 3, the IG scores for each of the sport nutrition topics are as follows: 46.3% protein/carbohydrate knowledge, 56.5% supplement knowledge, 62.0% timing of meals/ snacks and 82.6% hydration knowledge. Overall, the SNK scores of our participants (59.5%) were low compared to scores reported in the literature. The mean general nutrition or sport nutrition knowledge scores for HS athletes range from 55–74%, with higher scores in female athletes (65–68%) [38,39,40,41,53,61,62]. For females, SNK ranged from 65–68% in the research literature [38,40,41], and were similar to the scores for the females in our IG. Prior to our intervention, we measured SNK scores in a large cross-sectional sample of HS soccer players (*n* = 535) and found a mean SNK score of 45.6% [14] using the same SNK questionnaire [42]. A possible explanation for the lower than average SNK score (at baseline) among our IG athletes could be due to a higher proportion of females, who had lower SNK scores than our male athletes. Compared to studies in the research literature, our athletes were less elite, more diverse, and were participating in a team sport. Furthermore, this is the first study to use a diverse group of athletes in terms of ethnicity and income. Only Spendlove [62] has examined cross-sectional differences in ethnicity and general nutrition knowledge in athletes, but their athletes were more elite and older (mean age = 18.9 years) than the athletes in our study. Overall, 45% of our IG were Latino and 44% participated in NSLP. Little et al. [63] reported low general nutrition knowledge scores (~36%) in high school students (72% African American; 10% Latino) identified as NSLP participants. Neumark-Sztainer et al. [64] also reported that adolescents from low-SES backgrounds were at greater risk for inadequate food intake patterns. The sport nutrition questionnaire used in this study was validated using HS elite rugby players from Ireland [39], the only team sport to report SNK scores. This questionnaire was not tested for culture appropriateness with low-income or Latino populations. Although IG participants did not attain SNK scores as high as that reported in the research literature, a 10% increase in SNK suggests athletes are interested and engaged in learning sport nutrition information. In addition, we found a significant increase in athletes reporting to eat for performance (see below).

The majority of the intervention research in this area has examined changes in specific sport nutrition topics such as sport supplements [10,50] or hydration knowledge [52]. Studies examining comprehensive SNK (i.e., on topics such as timing of meals, hydration, use of supplements) provide few details on nutrition knowledge outcomes [10,55]. Both Philippou et al. and Nascimento et al. focused on individual adolescent athletes (12–19 years) vs. team sports and neither had a control or comparison group. After the intervention, Nascimento et al. [53] reported a SNK score of 84%, an 11% gain, while Philippou et al. [54] reported a median SNK score of 70% before and after the intervention. Another complication in comparing our SNK outcomes to other research studies is that there is no universally accepted SNK questionnaire for youth athletes.

Overall, research examining nutrition education interventions among HS athletes report positive changes in SNK [10,49,50,53,55], but that translation of this knowledge to changes in behaviors are typically not examined (discussed below). Future research should consider reporting details on the amount of change observed and using a standardized SNK questionnaire [65].

### 4.2. Changes in Dietary Attitudes/Beliefs Relevant to Sport Performance

In general, participants in the IG and CG agreed with the statement that ‘diet is important to performance’ (>92%). However, the groups varied in their belief that the nutritional requirements of active youth are different from their sedentary peers. Overall, the IG generally agreed that ‘as an athlete, my nutritional requirements are different’ (time 1 = 60.6%, time 2 = 68.7%, time 3 = 73.0%) compared to the CG, which ranged from 41–51% over the intervention. Across the intervention period, the IG participants reported an increase in ‘trying to eat for performance’ (time 1 = 41.8%; time 3 = 48.7%), while CG participants decreased this behavior (time 1 = 51.6%; time 3 = 30.2%). Overall, about 26–32% of the IG reported that ‘my diet meets my nutritional needs’, while 26% of the CG reported this belief at time 1, but by time 3 nearly half reported this belief (47.6%). These findings suggest that among the IG there was an increase in the awareness of the types of foods necessary to meet the needs of an adolescent athlete and a realization that their current diet plan was not adequate. This belief was translated into the athletes reporting that they were making an effort to eat the types and amounts of foods necessary for improving performance as evidence by a significant decrease in mean added sugar intake (12 g/day) in the IG [59]. These findings also support the multidimensionality of food choice among adolescents in general [66] and athletes in particular [67]. The gap between knowing what to include in a dietary plan and the actual foods consumed may be due to limited healthy food availability at home [68], school [66,69] or in the sport environment [16,70].

### 4.3. Dietary Behaviors Relevant to Sport Performance

Breakfast consumption is associated with increased consumption of key nutrients such as carbohydrate, protein, iron and calcium [34,71], which are important for adolescent growth and fueling physical activity [17]. In our study, nearly 50% of the all athletes reported consuming breakfast over the intervention, regardless of group assignment. This is lower than national data from the National Health and Nutrition Examination Study (1999–2006) that show 68% of 12–19 year-olds consume breakfast daily [72]. Although no changes over time were observed between the groups (IG vs. CG), it is important to note that all our participants, regardless of group assignment, were more likely to consume breakfast at baseline, than ‘non-completers’ (i.e., those participants who did not complete the full protocol). Compared to the participants who volunteered for the study initially (*n* = 535), completers (*n* = 217) were 1.5 times more likely (*p* = 0.041) to report eating breakfast daily at baseline (time 1) compared to non-completers. At time 1, fewer female participants (49%) reported eating breakfast daily compared to their male peers (73%) (OR 0.32, CI 0.16, 0.61, *p* = 0.001), with no program × gender interaction observed. The difference in daily breakfast consumption by sex was similar at time 3 (female = 49%, male = 63%; OR 0.52, CI = 0.28, 0.95, *p* = 0.033). Overall, there was no group difference (IG vs. CG) in breakfast consumption at time 3. No other differences by subpopulation (race/ethnicity or income) were observed.

Over the intervention, we observed significant changes in the consumption of lunch by participants. Overall, 92–94% of our IG reported ‘eating lunch 5 or more days per week’, while lunch consumption significantly decreased in the CG over intervention (time 1 = 98.4%; time 3 = 90.6%). These findings are similar to Tawfik et al. [73] who reported that 87.2% of their young Egyptian athletes (*n* = 312; 14.3 years) consuming lunch regularly. Research on the eating patterns of youth athletes (<18 years) is limited. Only Noll et al. (47) has examined this topic and reported equivocal results.

Sport nutrition recommendations for adolescent athletes include eating prior to physical activity and to initiate a recovery meal/snack soon after activity [17]. Our participants had soccer practice or games after school, which was typically 4 or more hours after lunch. Thus, we encouraged athletes to eat a snack and drink fluids prior to practice/games, since they would be at least 4 h post-prandial. Although IG participants who ate a snack prior to soccer practice increased by 10% over the intervention (time 1 = 33%; time 3 = 43%), this improvement was not statistically different from the CG. The improvement in snacking behavior among the IG participants prior to exercise was observed across all sub-populations (female = +15.8%; White = +13.2%; Latino = +7.7% and NSLP = +10.0%).

Overall, 72–78% of our athletes reported eating within 1 h after physical activity, with no significant changes due to the intervention. These findings differ from Nascimento et al. [53], who reported that their adolescent athletes (*n* = 11) had a 40% increase (pre = 40%; post = 80%) in food consumed before (1 h before) and after (1 h post) exercise. They provided athletes with four individualized nutrition counseling sessions based on personal dietary habits, needs and goals, over a 6-month period. Our sport nutrition intervention included 4 h of classroom sport nutrition education/experiential learning and 3 h of life-skills training (cooking, shopping, meal planning). All education was in a group setting and not individualized. Although coaches reported players started bringing healthy snacks to practice [56], our intervention did not mandate that team practices have designated ‘fluid/fueling breaks’. Specific environmental changes, such as ‘fluid breaks’ have successfully increased fluid consumption and improving hydration levels among HS athletes [51,52]. Cleary and colleagues [52] found that hydration education alone did not significantly influence water consumption during a summer athletic camp, until hydration needs were individualized and mandatory hydration breaks were given every 20 min. Similiarly, Kavouras et al. [51] used an adolescent athletic camp setting to provided environmental cues regarding hydration (urine color charts in restrooms; water bottles readily available throughout training and resting areas). They found that changes in the environment, plus a one-time educational intervention for players, coaches and parents, significantly increased fluid intake in intervention athletes compare to controls. Future research should examine the efficacy of individualized fueling intervention pre/post training, and encourage coaches to implement mandatory breaks for fueling/fluids during periods of training and competition in the fueling behavior of HS athletes.

### 4.4. Participant Engagement and Retention

The WAVE program began with 535 HS soccer players completing all baseline assessments [42] This manuscript examined only the participants (*n* = 271) who engaged at each of the intervention’s three assessment time points (baseline = time 1, time 2 = end year 1, time 3 = end year 2). We observed that participants who completed the full WAVE protocol were more likely to be assigned to the intervention group (70%), identify as female (64%), and report eating breakfast daily (58%) at baseline compared to participants who dropped out of the program or failed to complete all three assessment time-points (non-completers). Due to the recruitment of soccer teams vs. individual players, HS senior (12th grade) soccer players were in their final season of sport in year 1. Examination of baseline characteristics of “completers” showed that soccer players in their senior year (2% of sample) were less likely than freshman (9th grade) soccer players (50%) to complete the full WAVE protocol. Prior research by Nasciemento et al. [53] (2016) suggests that low adherence to nutrition intervention protocol may be due to schedule conflicts associated with training and daily routine. These authors note that only 50% of participants in their study completed all four consultations. They suggest that future research examine barriers faced by athletes to adopt healthy eating and participation in nutrition interventions.

### 4.5. Strengths and Limitations

Our study had several strengths. First, the sport nutrition education curriculum was developed and pilot tested by sport nutrition dietitians familiar with the research on adolescent athletes’ nutritional needs and areas of concern. The educational sessions, both in the pilot and the intervention, were also led by a sport nutrition dietitian with experience playing competitive soccer at the collegiate and professional levels. The sport nutrition curriculum, although give to HS soccer players, provides general sport nutrition education and could apply to all youth sports. Second, the TBW focused on life-skills to develop healthy food habits and was led by community health professionals trained in nutrition, cooking and gardening education. Third, our intervention was team-based, thus providing opportunity for team member reinforcement of the material outside of the lesson setting [56]. Fourth, we used a SNK questionnaire previously validated among adolescent team athletes [39]. Sixth, our study exams the changes in knowledge, attitudes/beliefs and behaviors before/during/after a 2-year intervention. This allows us to study the impact of the intervention over time while accounting for changes that may occur due to growth and maturation. Finally, the sample was diverse (sex, race/ethnicity and SES) allowing for examination of knowledge, attitudes/belief and behavior change differences by sub-populations.

This study also had limitations. First, the retention rate declined by 51% over the 2-year intervention due to participants graduating or dropping out of soccer. We recruited soccer teams for the WAVE intervention, which meant that some participants were in their final year of HS in Year 1. One approach to reduce dropout rates, while not sacrificing efficacy [74], would be to deliver the full curriculum (i.e., seven lessons) during one defined period (e.g., training sport camp, competitive season) rather than dividing lessons over two periods. In addition, the curriculum could be repeated/reinforced in subsequent periods (e.g., training sport camps, competitive seasons), and returning athletes could be recruited to assist with leading activities. Another concern in regard to retention of participants is that the IG was twice as likely to complete the full protocol compared to the CG. Although incentives (gift cards) and session reminders sent by mail or phone were made to all participants, the IG received face time with the WAVE project due to the SNK lessons and TBW. Offering a program to the CG that would increase contact time [75], without impacting the key aims of the project, could have strengthened the research design. Second, the teams were assigned into groups based on location (rather than randomized) to avoid cross contamination between teams during the 2-year period. Data analysis showed that team location did not impact retention. Third, we were unable to examine the impact of dose on changes in SNK in our IG. Dose is a multi-dimensional concept that typically includes contact frequency, length of exposure, duration of the intervention, and number of communication channels [76]. Although detailed participation in structured activities was recorded throughout the intervention (i.e., contact frequency), the data collection methods did not include assessment of multiple communication channels (e.g., teammate, coach, or parents). Peers [10], coaches [10,50,53] and parents [53,54] have been identified as key stakeholders within sport nutrition education. Assigning each stakeholder to roles within the nutrition education intervention while measuring the frequency of reinforcement by the stakeholder would strengthen our understanding of the communication channels and their influence on SNK and behaviors of the participants. 

## 5. Conclusions

The findings from this 2-year sport nutrition education and life-skills intervention among HS soccer players support past observations that HS sport teams are a viable adolescent target group for interventions to promote healthy eating behaviors. Overall, the intervention significantly improved total SNK scores, especially among female athletes, increased awareness of the nutritional needs of adolescent athletes and motivation to eat for performance, and encouraged eating behaviors related to daily lunch consumption. Changes over time among the IG were similar across the sub-population groups (sex, race/ethnicity, SES), with the exception of SNK, which was attributed to an increase among female IG participants. The maintenance of healthy dietary patterns among the study participants over the course of the intervention is encouraging, especially when prior research has highlighted the decline in breakfast and lunch consumption in this population. Skipping meals reduces the intake of key nutrients, such as calcium, iron, protein and carbohydrates, needed by growing active youth. Finally, the intervention significantly increased the athletes’ awareness that they have different nutritional needs from their non-active peers, and that their current diet plan failed to meet nutrition recommendations for active youth. Based on our experience, sport nutrition education interventions targeting team sport athletes should be paired with environmental interventions that increase access to healthy food options at school, home, and in the training/competition settings.

## Figures and Tables

**Table 1:** Key themes covered in the sport nutrition knowledge (SNK) questionnaire.^a.^

Theme	Topics Addressed by the Questions
**Training Schedule**	Position played, training schedule, hours of training during and outside school.
**Eating practices and behaviors around sport**	Typical eating patterns (breakfast, lunch, dinner, snacks); types of fluids consumed; timing of food/beverage intake before/after exercise; typical foods consumed.
**Attitudes and beliefs about food/nutrition relative to sport**	Importance of food/beverages consumed for sport performance; statements about diet that apply to them; nutritional needs of athletes are different from non-athlete peers.
**Sport Nutrition Knowledge**	**Hydration**—timing of fluid consumption; impact of dehydration on performance. **Pre/Post Exercise Food Selection**—foods consumed following sport training or game. Best food options 3–4 h prior to sport training and games and 1–2 h post-exercise training or game. **Protein/Carbohydrate Knowledge**—Timing and amount of carbohydrate and protein consumption around sport. Consequences if amounts are low; benefits if amounts are adequate. **Supplement Knowledge**—Accuracy of supplement claims regarding benefits; need for nutritional supplements.

^a^ The complete questionnaire can be found at Walsh, Cartwright et al. (2011).

**Table 2:** Sport nutrition lessons delivered face-to-face to High School Soccer players and their coaches.

Lesson	Title	Lesson Goals (30 Min Lessons)
**Year 1: Fall 2015**		
**1**	**Hydration**	To provide athletes with the knowledge to understand the purpose of proper hydration for sport and exercise, and give them the tools and skills needed to implement a hydration plan to delay onset of dehydration, improve and maintain training and performance, and decrease risk of illness and injury related to dehydration.
**2**	**Pre-Exercise Fueling**	To provide athletes with the knowledge necessary to understand the purpose of proper fueling prior to exercise, and give them the tools and skills necessary to implement a pre-exercise fueling plan to delay onset of fatigue and dehydration, improve and maintain training and performance, and avoid gastrointestinal (GI) discomfort.
**3**	**During Exercise Fueling**	To provide athletes with the knowledge to understand the purpose of proper fueling during exercise, and give them the tools and skills necessary to implement a during-exercise fueling plan to improve and maintain training and performance, and avoid GI discomfort.
**4**	**Recovery Nutrition**	To provide athletes with the knowledge to understand the purpose of proper recovery nutrition and the skills to implement a recovery nutrition plan. Key components included replete glycogen stores, rehydrate, initiate recovery and adaptation processes in the body that require carbohydrate, protein, and water, and achieve the maximum gains from training to maintain and/or improve performance.
**Year 2: Summer 2016**		
**5**	**Body Composition and Image**	Lessons were different for males and females. Males: To provide athletes with an understanding of body composition, how it is measured, and factors that influence body size and composition. Females: To provide athletes with an understanding of body composition and a heathy perspective on body image in order to develop body appreciation and acceptance.
**6**	**Maintaining Muscle and Staying Well**	To provide athletes with the knowledge to understand how nutrient timing and composition of foods/meals will help them meet their protein needs; maintain a strong immune system and stay healthy while participating in sports.
**7**	**Eating Well while Eating Out**	Help athletes understand how to make better food selections outside the home that are within their budgets. Raise awareness that cooking and eating food from home can be the most affordable and ‘healthy’ option.

**Table 3:** Characteristics of high school soccer players by group (intervention, comparison) and sex (male, female) at baseline (time 1).

	Total Sample (*n =* 217)	Intervention Group (IG) (*n =* 153)		Comparison Group (CG) (*n =* 64)	
	**Mean (SD)**				
		**Female**	**Male**	**Female**	**Male**
Height (cm)		161.8 (7.0)	171.2 (7.8)	160.1 (5.6)	172.8 (8.9)
Weight (kg) ^a^		58.7 (10.6)	62.9 (12.5)	59.9 (9.0)	68.2 (16.7)
Body Mass Index (kg/m^2^)		22.4 (3.6)	21.4 (3.7)	23.3 (3.1)	22.7 (5.2)
	**Total Sample (*n =* 217)**	**Intervention Group (IG) (*n =* 153)**		**Comparison Group (CG) (*n =* 64)**	
Age (y)	14.9 (0.91)	14.9 (0.91)		14.9 (0.91)	
Age preparing meals for self (y)	11.0 (2.1)	11.0 (2.1)		11.1 (2.2)	
Years playing soccer	6.9 (3.8)	7.3 (3.7)		5.8 (3.6) *	
	***n* size (%)**				
Sex					
Female	138 (64.0) **	94 (61.4)		44 (68.8)	
Male	79 (36.4)	59 (38.6)		20 (31.2)	
Race/Ethnicity *					
Latino	103 (47.5)	69 (45.1)		34 (53.1)	
White	96 (44.2)	61(44.4)		28 (43.8)	
Other ^b^	18 (8.3)	16 (10.5)		2 (3.1)	
Year in School					
9th grade	92 (42.4)	64 (42.1)		28(44.4)	
10th grade	63 (29.03)	47 (30.9)		16 (25.4)	
11th grade	56 (25.81)	39 (25.7)		17 (27)	
12th	4 (1.84) **	2 (1.3)		2 (3.2)	
No injuries past 12-months	142 (65.4)	97 (63.8)		45 (72.6)	
Participate in NSLP ^c^	100 (46.5)	67(44.4)		33 (51.6)	
Latino NSLP	82 (38.1)	56 (37.1)		26 (40.6)	
White NSLP	14 (6.5)	8 (5.3)		6 (9.4)	
Other NSLP	4 (1.9)	3 (2.0)		1 (1.6)	
Prepares meals for self (%)	56.9	55.9		59.4	

** Completers are statistically different (*p* ≤ 0.05) from incompleters using multiple logistic regression. * Groups are significantly different (<0.05) using *t*-test for variables reported in means and chi-squared tests for categorical variables, confidence at 95%. ^a^ Mean weight based on *n* = 284 due to missing data; ^b^ Other = African American, Asian-Pacific Islander, American Indian/Alaska Native; ^c^ NSAP = National School Lunch Program participation is used as an indicator of socioeconomic status.

**Table 4:** Sport nutrition knowledge (SNK) total score, domain scores and change scores at each time period (*n* = 217).

	Intervention Group (*n* = 153)				Comparison Group (*n* = 64)			
Variable	Time 1 ^a^	Time 2	Time 3	Change ^b^	Time 1	Time 2	Time 3	Change ^b^
Total SNK score	5.16 (1.80)	5.99 (1.93) **	6.09 (1.59)	0.93 ***	5.00 (1.62)	4.98 (1.79)	5.08 (1.38)	0.09
Hydration	2.27 (0.74)	2.41 (0.73) *	2.48 (0.64) **	0.21 **	2.19 (0.64)	2.17 (0.66)	2.19 (0.67)	0.00
Pre/Post Exercise Food Selection	0.42 (0.50) *	0.59 (0.49) **	0.62 (0.49) **	0.20 ***	0.27 (0.45)	0.34 (0.48)	0.35 (0.48)	0.08
Protein/Carbohydrate Knowledge	1.43 (0.96)	1.86 (0.93) **	1.85 (0.96) **	0.42 ***	1.21 (0.96)	1.38 (1.12)	1.44 (0.95)	0.21
Supplement Knowledge	1.04 (0.62)	1.09 (0.69)	1.13 (0.62)	0.09	1.19 (0.71)	1.10 (0.73)	1.10(0.64)	−0.08

^a^ Independent *t*-test IG compared to CG at same time period: *p ≤ 0.05; ** *p* ≤ 0.010; *** *p* ≤ 0.001. ^b^ Paired *t*-test comparing mean score at time 3 and time 1 within treatment group: * *p* ≤ 0.05; ** *p* ≤ 0.010; *** *p* ≤ 0.001.

**Table 5:** Odds ratios (95% CI) of self-reported attitudes and beliefs relevant to sport nutrition over time (Time 1–3) and overall.

	Time 1			Time 2			Time 3		
	Comparison *n* (%)	Intervention *n* (%) ^a^	OR ^b^	Comparison *n* (%)	Intervention *n* (%) ^a^	OR (95% CI; *p*-value)	Comparison *n* (%)	Intervention *n* (%) ^a^	OR (95% CI; *p*-Value)
Diet is important to performance	56 (88.9)	120 (84.5)	1.00	59 (92.3)	132 (88.0)	0.92 (0.27, 3.16); 0.90	59 (93.6)	140 (92.1)	1.15 (0.30, 4.34); 0.83
As an athlete, my nutritional requirements are different	26 (41.3)	86 (60.6) **	1.00	30 (46.9)	103 (68.7) **	1.27 (0.64, 2.52), 0.50	33 (51.6)	111 (73.0) **	1.23 (0.62, 2.49); 0.55
I have trouble knowing what to eat	14 (22.6)	43 (30.5)	1.00	15 (23.8)	33 (22.0)	0.61 (0.25, 1.49); 0.28	14 (22.2)	26 (17.1)	0.50 (0.20, 1.25); 0.14
My eating plan/diet meets my nutritional requirements	16 (25.8)	37 (26.2)	1.00	26 (41.3)	46 (30.7)	0.57 (0.24, 1.31); 0.19	30 (47.6)	48 (31.6) *	0.43 (0.18, 0.99); ***0.05***
Muscle mass is important to my performance	33 (52.4)	97 (68.3) *	1.00	32 (50.0)	106 (70.7) **	1.26 (0.62, 2.56); 0.52	32 (50.0)	94 (62.2)	0.95 (0.47, 1.92); 0.83
Nutritional supplements are necessary to support my training	35 (44.6)	71 (49.6)	1.00	24 (37.5)	64 (42.7)	1.82 (0.90, 3.71); 0.10	25 (39.1)	65 (42.8)	1.82 (0.89, 3.71); 0.10
I try to eat for performance	32 (51.6)	59 (41.8)	1.00	21 (33.3)	69 (46.0)	2.62 (1.20, 5.70); ***0.02***	19 (30.2)	74 (48.68) *	3.51 (1.59, 7.77); ***0.02***

**^a^** X^2^ test of independence IG compared to CG at time point; * *p* ≤ 0.05; ** *p* ≤ 0.010; **^b^** Time 1 is the reference category when examining change in outcome variable by program type over time using results from logistic regression analysis of longitudinal data fit by generalized estimating equations.

**Table 6:** Odds ratios (95% CI) of self-reported behaviors relevant to sport nutrition over time (Times 1, 2 and 3) and overall.

	Time 1			Time 2			Time 3		
	Comparison *n* (%)	Intervention *n* (%) ^a^	OR ^b^	Comparison *n* (%)	Intervention *n* (%) ^a^	OR (95% CI; *p*-Value)	Comparison *n* (%)	Intervention *n* (%) ^a^	OR (95% CI; *p*-Value)
Eat breakfast every day	35 (55.6)	83 (58.4)	1.00	31 (48.4)	81 (54.0)	1.14 (0.62, 2.10); 0.67	34 (53.1)	82 (54.0)	0.90 (0.49, 1.64); 0.72
Eat lunch 5 or more days a week	61 (98.4)	130 (92.2)	1.00	56 (87.5)	139 (92.7) *	9.97 (1.30, 76.6); ***0.27***	58 (90.6)	142 (93.4) *	8.62 (1.10, 67.57); ***0.04***
Eat within 1 h before physical activity (PA)	24 (38.7)	47 (33.1)	1.00	26 (41.3)	49 (33.1)	0.84 (0.41, 1.73); 0.65	21 (33.3)	66 (43.7)	1.89 (0.91, 3.91); 0.09
Eat within 1 h after PA	46 (71.8)	118 (77.1)	1.00	52 (81.2)	126 (82.4)	0.73 (0.29, 1.81); 0.50	51 (79.7)	117 (76.5)	0.58 (0.24, 1.41); 0.23
Consumption of any sugar sweetened beverage 1–4 h before PA	33 (51.6)	105 (68.6) *	1.00	34 (53.1)	97 (63.4)	0.75 (0.36, 1.55); 0.44	26 (40.6)	79 (51.6)	0.75 (0.36, 1.58); 0.45

**^a^** X^2^ test of independence IG compared to CG at time point; * *p* ≤ 0.05; **^b^** Time 1 is the reference category when examining change in outcome variable by program type over time using results from logistic regression analysis of longitudinal data fit by generalized estimating equations.

## Supplementary Materials

The following are available online at http://www.mdpi.com/2072-6643/10/11/1636/s1, Figure S1: The WAVE program intervention experimental design (2015–2017).
